# Relation between depression and sociodemographic factors

**DOI:** 10.1186/1752-4458-1-4

**Published:** 2007-09-04

**Authors:** Noori Akhtar-Danesh, Janet Landeen

**Affiliations:** 1 School of Nursing and Department of Clinical Epidemiology and Biostatistics, McMaster University, Hamilton, Canada; 2 School of Nursing and Department of Psychiatry & Behavioural Neurosciences, McMaster University, Hamilton, Canada

## Abstract

**Background:**

Depression is one of the most common mental disorders in Western countries and is related to increased morbidity and mortality from medical conditions and decreased quality of life. The sociodemographic factors of age, gender, marital status, education, immigrant status, and income have consistently been identified as important factors in explaining the variability in depression prevalence rates. This study evaluates the relationship between depression and these sociodemographic factors in the province of Ontario in Canada using the Canadian Community Health Survey, Cycle 1.2 (CCHS-1.2) dataset.

**Methods:**

The CCHS-1.2 survey classified depression into *lifetime depression *and *12-month depression*. The data were collected based on unequal sampling probabilities to ensure adequate representation of young persons (15 to 24) and seniors (65 and over). The sampling weights were used to estimate the prevalence of depression in each subgroup of the population. The multiple logistic regression technique was used to estimate the odds ratio of depression for each sociodemographic factor.

**Results:**

The odds ratio of depression for men compared with women is about 0.60. The lowest and highest rates of depression are seen among people living with their married partners and divorced individuals, respectively. Prevalence of depression among people who live with common-law partners is similar to rates of depression among separated and divorced individuals. The lowest and highest rates of depression based on the level of education is seen among individuals with less than secondary school and those with "other post-secondary" education, respectively. Prevalence of 12-month and lifetime depression among individuals who were born in Canada is higher compared to Canadian residents who immigrated to Canada irrespective of gender. There is an inverse relation between income and the prevalence of depression (p < 0.0001).

**Conclusion:**

The patterns uncovered in this dataset are consistent with previously reported prevalence rates for Canada and other Western countries. The negative relation between age and depression after adjusting for some sociodemographic factors is consistent with some previous findings and contrasts with some older findings that the relation between age and depression is U-shaped. The rate of depression among individuals living common-law is similar to that of separated and divorced individuals, not married individuals, with whom they are most often grouped in other studies.

## Background

Depression is a significant public health concern worldwide and has been ranked as one of the illnesses having the greatest burden for individuals, families, and society [1,2]. In Canada depression accounts for $14.4 billion annually of health care spending, lost productivity, and premature death [3]. As well, depression is related to increased morbidity and mortality from medical conditions [4-6] and decreased quality of life [7,8] among many other negative consequences.

Given the significant impact of depression on individuals and society as a whole, a comprehensive analysis of the prevalence of depression is necessary to ensure that previous findings remain applicable in today's society. The most recent national data comes from the Canadian Community Health Survey: Mental Health and Well-being (CCHS 1.2) which has found a lifetime prevalence for major depression in 12.2% and major depression occurring in the past 12 months in 4.8% of the population [9]. This is consistent with earlier epidemiological studies of depression in Canada which have found one-year prevalence rates ranging from 4 to 12% [9-11] similar to findings from the United States [12,13]. Demographics of Canada, and particularly of Ontario, are changing with the aging population and the increasing numbers of new immigrants. Recent statistics shows that Ontario receives more than 50 percent of immigrants to Canada [14]. Based on regional changes in the demographic characteristics of the population, it is important to examine regional subsets of the Canadian data on depression.

The sociodemographic factors of age, gender, marital status, education, and income have consistently been identified as important factors in explaining the variability in the prevalence of depression. Key North American studies, particularly the Epidemiologic Catchment Area Study [13], the National Comorbidity Survey [15], the Canadian National Population Health Survey [16], and the Ontario Health Survey [10] found prevalence rates varying from 2.8% to 10.3%, based on the demographic factors of age and gender. Patten and colleagues [9] report that the CCHS 1.2 Canadian-wide survey found significant interactions among age, sex, and marital status, with single women reporting lower rates of depression with increased age and single men reporting increasing rates [9]. It is important for local planners to have a more detailed analysis of the picture of depression in Ontario.

Previous research has found that age is one of the demographic characteristics that accounts for much of the variance in the prevalence of depression. A Canadian National Population Health Survey found that the prevalence of 12-month depression varied in men from "too low to report" for men over 65 to a high of 5.2% for the 12 to 24 age group [16]. Women's prevalence also varied by age, ranging from a low of 3.1% for women over 65 to a high of 9.6% for the 12 to 24 age group [16]. The Ontario Health Survey found comparable variation based on age [10]. This pattern is consistent with findings from Australia [17].

Prevalence for depression has also been found to vary considerably based on gender [18]. Consistently, women have nearly double to triple the prevalence rates for 12-month depression compared to men [10,15-17,19]. There are also gender differences in both the use of outpatient treatment [12] and response to antidepressants [20].

Marital status has been found to interact with gender in accounting for variance in the prevalence of depression. In Australia, those who were separated or divorced had a high rate of anxiety disorders (18%) and affective disorders (12%) [17]. In Canada, single mothers have been found to have prevalence of 15.4% compared to 6.8% for married mothers [21], although this increase in rate of depression may relate to the demands of parenting rather than on marital status, per se.

Traditional wisdom has long held that there is an association between depression and socioeconomic status (SES). Several recent studies confirm a strong inverse relationship between SES and mental disorder [18,22-24]. Published research indicates that despite differences in definitions and measurements of SES, the likelihood of depression in the lowest SES group is as much as twice that found in the highest SES group [24,25]. People in the lowest class are far more likely to suffer from psychiatric distress than those in the highest class [26]. Lennon et al. [23] concluded that one out of every five women on welfare met standard criteria for major depression. Epidemiological studies of depression in Canada and United States found differences in the prevalence rates of depression based on SES factors [10-13,27]. However, a review by Kohn et al. [28] found that patterns of relationships were not always consistent. Therefore, it is prudent to periodically reassess the relationship between depression and sociodemographic factors because of the changing demographic composition of Ontario.

While effective services and treatments for depression have been identified, the stigma associated with depression has been identified as a barrier to seeking treatment [29]. Worldwide stigma and discrimination have been recognized as major contributors to increasing the burden of mental illness and negative attitudes towards mental illness have been distressingly pervasive [30]. It is known that attitudes toward mental illness (including stigma and discrimination) vary across cultures, and symptoms may also vary while the underlying illness remains the same [30,31]. The cultural mosaic of Canada, and particularly Ontario, is changing as all population growth within the region is now attributable to immigration as the birth rate declines [32]. Thus, it is important to begin to explore the rate of depression in the immigrant population of Ontario. Examining the relationships among the prevalence of depression, immigration status, and demographic factors for the changing population in Ontario is a relevant first step in unraveling some the complex interactions for this significant problem.

In this article we use the dataset from the 2002 Canadian Community Health Survey, Cycle 1.2 (CCHS-1.2) to estimate the prevalence rate of depression in Ontario and whether or not there are differences in subgroups of the population based on the sociodemographic factors of age, gender, marital status, immigration status, education, and income level.

## Methods

The Canadian Community Health Survey classified depression into *lifetime depression *and *12-month depression*. One strength of the CCHS 1.2 survey is that it used the Composite International Diagnostic Interview (CIDI) developed by the World Mental Health Project to measure depression [33] with major refinements from the original CIDI, although limited validation studies have been published on the tool [9]. The CIDI was designed to capture cross cultural incidence of mental illness. However, there have been some concerns about the potential for misunderstanding of key concepts used in the survey which might result in an under-reporting from individuals with low education [33]. Every attempt was made to minimize language bias, with interviewers recruited "with a wide range of language competencies. To help these interviewers, an 'official' translation of key terms was created in Chinese and Punjabi, the two most prevalent non-official languages from CCHS Cycle 1.1. Interviewers were restricted from conducting interviews in any other language because of the complexity of the question concepts. Cultural biases toward mental illness could also have led to an under-reporting of depression among immigrant groups.

Respondents who experienced the following criteria associated with major depressive episode (MDE) were classified as being affected by *lifetime depression*: 1) a period of two weeks or more with depressed mood or loss of interest or pleasure and at least five additional symptoms from the following nine: depressed mood, diminished interest in hobbies or activities, significant weight loss/gain or change in appetite, insomnia or hypersomnia, psychomotor agitation or retardation, fatigue or loss of energy, feelings of worthlessness, diminished ability to think or concentrate, and recurrent thoughts of death, 2) clinically significant distress or social or occupational impairment; and, 3) the symptoms are not better accounted for by bereavement.

Also, respondents who experienced the following criteria associated with MDE were classified as having *12-month depression*, 1) meet the criteria for lifetime diagnosis of MDE, 2) report a 12-month episode, and 3) report marked impairment in occupational or social functioning. These definitions are consistent with the classifications of major depression found in the DSM-IV [34]. The standard algorithm for establishing the existence of depression on the CIDI was used and no further restrictions were used except for those indicated above. The additional requirement of meeting clinical significance was not noted in the CCHS 1.2, which has been suggested to minimize any potential over-reporting of mental disorders using the CIDI [33,35].

### Data Source

The CCHS-1.2 dataset which includes 12376 respondents in Ontario is based on unequal sampling probabilities due to the design of the study to ensure adequate representation of the sample. One person aged 15 and over was randomly selected from each sampled household. Individuals living in health care institutions, in the military, or living on Indian Reserves were excluded from the survey.

### Statistical Analysis

To control for the non-proportional sampling effect of the CCHS-1.2 dataset the proper sampling weights provided by Statistics Canada were used to calculate the percentages of participants in each subgroup of the population and to estimate the prevalence rates of depression. Then, for each prevalence rate, a 95 percent confidence interval (95%CI) is provided using the bootstrap re-sampling program provided by Statistics Canada. Also, the bootstrap program was used in a multiple logistic regression technique to estimate the odds ratio of depression for each demographic and socio-economic factor. The sampling weights were used in conducting chi-square tests and chi-square test for trend. The statistical program SPSS version 15 was used for statistical analysis.

## Results

Among the 12376 participants there were 5660 males and 6716 females. Table 1 represents the number and percentages of participants based on the sociodemographic factors. About 64% of the participants were 25–64 years old and 57.1% of them were married and living with their spouses at the time of participation. About 32 percent of the participants immigrated to Canada compared to 68% who were born in Canada. Most of the participants had some post-secondary education (56.5%) and about 40.0% of the participants lived with annual household income of less than $50,000.

**Table 1:** Distribution of the sociodemographic variables in the sample

	**Male**		**Female**		**Total**	
	Number	Percent	Number	Percent	Number	Percent

**Age group (Year)**						
15–19	508	10.0	490	8.5	998	9.2
20–24	391	7.0	500	7.2	891	7.1
25–44	2089	40.0	2345	38.5	4434	39.0
45–64	1692	29.7	1780	29.5	3472	29.6
65–74	570	8.4	848	9.6	1418	9.0
≥75	410	5.0	753	6.8	1163	5.9

**Total**	**5660**	**100.0**	**6716**	**100.0**	**12376**	**100.0**

**Marital status**						
Married	2835	58.0	2984	56.2	5819	57.1
Common-law	352	6.7	356	5.1	708	5.9
Widowed	251	2.3	994	8.1	1245	5.2
Separated	219	2.2	300	3.0	519	2.6
Divorced	332	3.2	516	5.1	848	4.2
Single	1665	27.6	1558	22.6	3223	25.0

**Total**	**5654**	**100.0**	**6708**	**100.0**	**12362^§^**	**100.0**

**Education**						
Less than secondary	1417	23.5	1797	24.1	3214	23.8
Secondary graduation	1029	18.0	1309	21.4	2334	19.7
Other post-secondary	491	8.9	586	8.6	1077	8.8
Post-sec graduation	2688	49.6	2990	45.9	5678	47.7

**Total**	**5625**	**100.0**	**6682**	**100.0**	**12307^§^**	**100.0**

**Immigrant**						
Yes	1297	32.2	1601	31.6	2898	31.9
No	4339	67.8	5078	68.4	9417	68.1

**Total**	5636	100	6679	100	12315	100

**Household income**						
< $10,000	162	2.0	235	2.3	397	2.2
$10,000–$14,999	214	2.4	527	4.7	741	3.6
$15,000–$19,999	192	2.8	392	4.2	584	3.5
$20,000–$29,999	518	7.5	793	9.7	1311	8.6
$30,000–$39,999	593	10.1	758	11.3	1351	10.7
$40,000–$49,999	562	9.5	608	10.2	1170	9.8
$50,000–$59,999	536	10.1	576	10.8	1112	10.5
$60,000–$79,999	955	18.8	922	17.4	1877	18.1
$80,000 or more	1554	36.7	1302	29.3	2856	33.0

**Total**	**5286**	**100.0**	**6113**	**100.0**	**11399^§^**	**100.0**

**§ **The total number is different from 12376 because of missing or non-applicable data

In total, the prevalence rates of *lifetime depression *and *12-month depression *are 11.0% (95%CI, 10.2 to 11.7) and 4.8% (95%CI, 4.3 to 5.3) in the province of Ontario. Table 2 presents the prevalence rate of lifetime and 12-month depression based on the sociodemographic factors.

**Table 2:** Prevalence of lifetime and 12-month depression based on the sociodemographic factors

	**Lifetime depression**					**12-month depression**				
	**Gender**		**Total**			**Gender**		**Total**		
	
**Demographic factor**	**Male**	**Female**	**(%)**	**95%CI**	***χ*^2 ^(P-value)**	**Male**	**Female**	**(%)**	**95%CI**	***χ*^2 ^(P-value)**

**Age**										
15–19	3.1	12.1	7.4	(5.1, 9.6)	138.980 (<0.001)	1.9	9.6	5.5	(3.5, 7.5)	104.879 (<.001)
20–24	11.1	17.3	14.3	(11.3, 17.3)		7.5	11.5	9.6	(7.0, 12.2)	
25–44	9.1	15.1	12.1	(10.9, 13.3)		3.6	6.6	5.1	(4.3, 5.9)	
45–64	9.8	15.3	12.6	(11.0, 14.2)		3.7	5.4	4.6	(3.5, 5.7)	
65–74	4.5	7.8	6.3	(4.8, 7.7)		1.6	2.1	1.8	(1.1, 2.6)	
≥75	3.6	4.8	4.3	(2.9, 5.7)		1.6	1.7	1.7	(0.8, 2.5)	

**Marital status**										
Now married	6.0	11.0	8.5	(7.6, 9.5)	301.652	2.0	3.5	2.8	(2.3, 3.3)	
Common-law	18.4	22.3	20.2	(15.4, 25.0)		9.4	10.8	10.0	(5.7, 14.2)	
Widowed	8.8	10.6	10.2	(8.0, 12.0)	(<.001)	4.7	4.3	4.4	(2.8, 6.0)	243.295
Separated	22.6	25.2	24.2	(19.2, 29.2)		10.4	15.4	13.4	(9.4, 17.4)	(<.001)
Divorced	22.7	27.7	26.1	(21.9, 30.2)		7.6	14.1	11.7	(8.6, 14.7)	
Single	7.3	14.7	10.7	(9.3, 12.2)		3.9	9.2	6.3	(5.1, 7.6)	

**Immigrant**										
Yes	6.5	9.1	7.8	(6.3, 9.4)	60.699	3.2	4.8	4.0	(3.0, 5.0)	**19.619**
No	9.1	15.8	12.4	(11.5, 13.3)	(<.001)	3.6	6.6	5.4	(4.5, 5.7)	(<.001)

**Education**										
< Secondary	7.1	11.1	9.1	(7.7, 10.6)	23.241 (<0.001)	3.7	6.3	5.0	(3.9, 6.1)	9.586 (=.022)
Secondary grad.	8.8	12.1	10.6	(8.9, 12.3)		3.0	5.2	4.2	(3.2, 5.2)	
Other post-sec.	11.1	15.6	13.4	(10.5, 16.2)		6.1	6.5	6.3	(4.4, 8.2)	
Post-sec. grad.	8.0	15.2	11.5	(10.4, 12.6)		3.1	6.3	4.7	(3.9, 5.5)	

**Household**										
**income**										
<$10,000	16.9	19.7	18.4	(13.4, 23.4)	χTrend2 MathType@MTEF@5@5@+=feaafiart1ev1aaatCvAUfKttLearuWrP9MDH5MBPbIqV92AaeXatLxBI9gBaebbnrfifHhDYfgasaacH8akY=wiFfYdH8Gipec8Eeeu0xXdbba9frFj0=OqFfea0dXdd9vqai=hGuQ8kuc9pgc9s8qqaq=dirpe0xb9q8qiLsFr0=vr0=vr0dc8meaabaqaciaacaGaaeqabaqabeGadaaakeaaiiGacqWFhpWydaqhaaWcbaGaemivaqLaemOCaiNaemyzauMaemOBa4MaemizaqgabaGaeGOmaidaaaaa@3630@ = 51.24 (<.0001)	9.3	13.1	11.3	(7.3, 15.3)	χTrend2 MathType@MTEF@5@5@+=feaafiart1ev1aaatCvAUfKttLearuWrP9MDH5MBPbIqV92AaeXatLxBI9gBaebbnrfifHhDYfgasaacH8akY=wiFfYdH8Gipec8Eeeu0xXdbba9frFj0=OqFfea0dXdd9vqai=hGuQ8kuc9pgc9s8qqaq=dirpe0xb9q8qiLsFr0=vr0=vr0dc8meaabaqaciaacaGaaeqabaqabeGadaaakeaaiiGacqWFhpWydaqhaaWcbaGaemivaqLaemOCaiNaemyzauMaemOBa4MaemizaqgabaGaeGOmaidaaaaa@3630@ = 103.08 (<.0001)
$10,000–$14,999	11.2	18.5	16.1	(13.0, 19.1)		6.2	10.2	8.9	(6.5, 11.3)	
$15,000–$19,999	13.0	17.5	15.7	(11.4, 19.9)		8.5	9.2	8.9	(5.9, 12.0)	
$20,000–$29,999	8.4	13.5	11.3	(9.2, 13.4)		4.5	7.2	6.0	(4.4, 7.7)	
$30,000–$39,999	8.0	11.9	10.1	(8.0, 12.2)		2.8	5.5	4.3	(2.9, 5.6)	
$40,000–$49,999	9.0	12.1	10.6	(8.4, 12.8)		3.3	4.1	3.7	(2.4, 5.0)	
$50,000–$59,999	7.5	13.4	10.5	(8.2, 12.9)		3.4	4.2	3.8	(2.3, 5.3)	
$60,000–$79,999	7.0	12.7	9.7	(8.2, 11.3)		2.8	5.1	3.9	(2.9, 5.0)	
≥$80,000	7.9	14.0	10.6	(9.1, 12.2)		2.8	4.9	3.8	(2.7, 4.9)	

**Total**	**8.2**	**13.7**	**11.0**	**(10.2, 11.7)**		**3.5**	**6.1**	**4.8**	**(4.3, 5.3)**	

### Depression and age

The highest prevalence rate of lifetime depression (14.3%) is seen in the age group of 20 to 24 years and the lowest rate (4.3%) in the age group of 75 years and over. Similarly, the highest and lowest rates of 12-month depression are 9.6% and 1.7%, which are seen in the same age groups (Table 2). The prevalence of both types of depression increases with age to the highest level for 20 to 24 year olds and then decreases steadily to its lowest level for the participants aged 75 years and over.

### Depression and gender

Women suffer more from both types of depression than men. The highest and lowest rates of lifetime and 12-month depression for both men and women were recorded in the age groups of 20 to 24 years and 75 and over, respectively (Table 2).

### Depression and marital status

The prevalence of depression varies with the marital status. The highest rates of lifetime and 12-month depression are seen in divorced and separated respondents, respectively. The lowest rate for both types of depression is seen among married people. Also, a high rate is seen among individuals living with "common-law" partners where the term "common-law" refers to the living of a man and a woman together in a marital status without legal action (Table 2).

### Depression and Immigrant Status

In general prevalence of depression among individuals who were born in Canada is higher compared to Canadian residents who immigrated to Canada irrespective of gender and type of depression (Table 2).

### Depression and education

Respondents whose education level was less than secondary school have the lowest rate of lifetime depression (9.1%); and the highest rate of lifetime depression (13.4%) is seen among those with "other post-secondary" education. A similar pattern is seen for 12-month depression. Although these results indicate that the prevalence of depression differs based on the level of education, there is no linear pattern for this relationship. For both lifetime and 12-month depression the prevalence rate was higher for "other post secondary education" than "post-secondary education".

### Depression and household income level

The highest prevalence rate of lifetime depression (18.4%) is seen in households with an income level of less than $10,000 per year. The prevalence of lifetime depression then decreases as the income increases. The same pattern is observed for 12-month depression with the highest rate of 11.3% in households with the income of less than $10,000 per year. However, there seems to be an threshold effect as the prevalence rate decreases much faster for income level of up to $30,000 than for $30,000 and over which will be further elaborated in the modeling section. The *chi-square test for trend *indicates that for both types of depression there is a noticeable inverse relationship between the level of income and the prevalence of depression (p < 0.0001; Table 2).

### Modeling depression based on the sociodemographic factors

We performed a logistic regression analysis for lifetime and 12-month depression to identify the most important sociodemographic factors associated with depression. Gender and marital status were considered as categorical variables with "female" and "single" as reference groups for gender and marital status, respectively. Education was dealt with as a categorical variable while "other post secondary education" and "post-secondary education" were combined into one group and the "less than secondary education" group was considered as the reference group. The immigrant status was categorized as 'yes' and 'no' identifying who immigrated to Canada and those who were born in Canada (the reference group). Table 2 indicates a strong inverse relation between household income and depression up to income level of $30,000. Then, the prevalence rate becomes approximately stable. Based on this finding we classified the income level of $30,000 and more into one category for the modeling purposes. The following noticeable results emerged from this analysis (Table 3):

**Table 3:** Relationship between depression and the sociodemographic factors

**Variable**	**B**	**SE (B)**	**P-value**	**OR^‡^**	**95% CI for OR**	
					
					**Lower**	**Upper**
**A: Lifetime depression**						

Age/10^§^	-0.10	0.03	<0.0001	0.90	0.86	0.95
Gender						
Female^†^				1.00		
Male	-0.53	0.09	<0.0001	0.59	0.50	0.70
Marital Status						
Single^†^				1.00		
Married	0.00	0.12	0.998	1.00	0.78	1.28
Common-law	0.90	0.18	<0.0001	2.46	1.73	3.51
Widowed	0.26	0.19	0.182	1.30	0.89	1.90
Separated	0.97	0.17	<0.0001	2.65	1.91	3.67
Divorced	1.20	0.15	<0.0001	3.33	2.46	4.50
Education						
Less than secondary^†^				1.00		
Secondary	0.23	0.14	0.096	1.26	0.96	1.66
Post-secondary	0.43	0.12	0.0003	1.54	1.22	1.93
Household income	-0.20	0.14	<0.0001	0.82	0.74	0.90
Immigrant status (yes, no)	-0.52	0.14	0.0001	0.60	0.46	0.78
Constant	-1.05	0.23	<0.0001	0.35	0.22	0.55

**B: 12-month depression**						

Age/10^§^	-0.19	0.05	<0.0001	0.83	0.76	0.90
Gender						
Female^†^				1.00		
Male	-0.46	0.14	0.0008	0.63	0.48	0.83
Marital Status						
Single^†^				1.00		
Married	-0.36	0.19	0.0651	0.70	0.48	1.02
Common-law	0.89	0.28	0.0016	2.43	1.40	4.23
Widowed	0.18	0.32	0.5713	1.20	0.64	2.24
Separated	1.16	0.23	<0.0001	3.20	2.05	4.97
Divorced	1.01	0.23	<0.0001	2.76	1.75	4.33
Education						
Less than secondary^†^				1.00		
Secondary	0.06	0.19	0.753	1.06	0.73	1.55
Post-secondary	0.30	0.17	0.073	1.35	0.97	1.86
Household income	-0.35	0.59	<0.0001	0.71	0.63	0.79
Immigrant status (yes, no)	-0.19	0.19	0.3266	0.83	0.57	1.20
Constant	-0.99	0.32	0.0022	-	0.19	0.70

‡ Odds ratio, § Age divided by 10, † Reference group

1. The odds of being affected by lifetime and 12-month depression for men is about 0.60 times of that for women.

2. Marital status emerged as an important predictor for both types of depression:

a. The odds of being affected by lifetime depression for married persons is about the same as that for single individuals. For 12-month depression the odds ratio is 0.70, although it is not significantly different from one (95%CI, 0.48 to 1.02).

b. The odds of lifetime or 12-month depression among individuals who live with common-law partners is about 2.5 times of that for singles.

c. The odds of being affected by lifetime depression for separated and divorced individuals is more than 2.5 times of that for singles; odds ratio is 2.65 (95%CI, 1.91 to 3.67) for separated and 3.33 (95%CI, 2.46 to 4.50) for divorced individuals. For 12-month depression these odds ratios are 3.20 (95%CI, 2.05 to 4.97) and 2.76 (95%CI, 1.75 to 4.33), respectively.

3. The odds of living with lifetime depression among individuals with any kind of post-secondary education is 1.54 times compared to individuals with less than secondary education (95%CI: 1.22 to 1.93). For 12-month depression this odds is not statistically significant (odds ratio = 1.35; 95%CI: 0.97 to 1.86).

4. Income has a significant association with both lifetime and 12-month depression. For the income level of up to $30,000 the odds ratio of lifetime depression for each $10000 increase in income is 0.82 (95%CI, 0.74 to 0.90). Similarly, for 12-month depression the odds ratio is 0.71 (95%CI, 0.63 to 0.79).

5. Prevalence rate of lifetime depression among immigrants is 60% of that among Canadian born individuals. For the 12-month depression the odds ratio is 0.83 but it is not statistically significant (95%CI, 0.57 to 1.20).

## Discussion

The findings of this analysis illustrate that despite changes in the overall demographics of the Ontario population, the prevalence rates of depression remain consistent with previous Canadian and American epidemiological surveys [10,15]. The patterns uncovered in this analysis are at the higher limits of the previously reported prevalence rates for Canada, and in particular for the province of Ontario [10]. The rates for depression in Ontario are consistent with the results from the larger Canadian sample from which this dataset has been extracted.[9]. This finding was anticipated given that Ontario represents about 40% of the total Canadian population [36]. This study has further described current distribution of depression based on age, gender, marital status, education, immigrant- nonimmigrant status, and household income.

Overall, our findings are consistent with some previous findings that women have double to triple the prevalence rates for 12-month depression compared to men [10,15,17,19], however, the age-specific rates in Table 2 indicate that gender differences diminish with age and there is virtually no difference at age 75+ which is in agreement with Gutiérrez-Lobos et al. [37].

We observed a negative relation between age and depression for both lifetime and 12-month depression after adjusting for some sociodemographic factors using logistic regression technique. These results are consistent with some previous findings [38-40] which rule out some older findings that relation between age and depression is U-shaped with the lowest reported levels of depression at ages 45–49 [41,42].

The relation between depression and marital status is highly significant. While our analysis confirms previous reported patterns for depression based on marital status [43], one notable difference emerged. The prevalence of depression in individuals living common-law was similar to that of separated and divorced individuals, not married individuals with whom they are most often grouped in other studies, including the recent report using the same dataset for all Canada [9]. This finding challenges the common practice of combining married and common-law individuals in the same category and suggests that these groups should be analyzed separately until the consistency of this finding is upheld or refuted.

While rates of depression in individuals born in Canada were higher than for immigrants, depression is still a health concern for the immigrant group. We found that immigrant status was highly related to lifetime depression but not to depression in the past year. Because this was a cross-sectional survey it is impossible to know if the depression occurred before or after immigration. Furthermore, the only data available on immigrant status was whether or not the individual was born in Canada. While attempts were made to minimize language barriers for data collection, it would be very helpful to know whether the respondents were refuges or skilled immigrants and how recently they immigrated. This survey only provides the starting point for exploring depression in immigrants in Ontario, and future studies are required to gain a better understanding of the complexities of factors that may contribute to depression in this group. Furthermore, given the cross-cultural variation in attitudes toward depression [44], the findings may not reflect accurate depression rates amongst recent immigrants.

Socioeconomic status, as indicated by education and income, also showed significant association with depression. In a multivariate analysis using logistic regression analysis, these variables showed strong relationships with depression.

Nevertheless, it remains important to target programs for those at the lowest income level, particularly to women between the ages of 20 and 64 and people who are divorced or separated. In the light of this analysis, individuals who live with "common-law" partners need special attention.

In conclusion, this study has provided a new snapshot of the prevalence of depression in Ontario. While this is not very different from what has been found before, we have provided details of subgroups of the population who are most at risk for depression. These findings are potentially important as they are from a large random sample of respondents. The results show significant relationships between depression and different sociodemographic factors. The results confirm the findings from other Canadian and international studies [10,11] and add strong weight toward the confirmation of such relations.

One limitation of the CCHS-1.2 dataset might be that it provides self-reported information from a cross-sectional study. Furthermore, because of funding and space limitations, we analyzed only Ontario data, and replicating this analysis in all of Canada would be beneficial to health policy makers.

## Competing interests

The author(s) declare that they have no competing interests.

## Authors' contributions

Both authors contributed equally in designing the study and drafting the manuscript. The analysis was carried out by NAD. Both authors read and approved the final manuscript.

## References

[B1] U.S. Department of Health and Human Services (1999). Mental health: A report of the surgeon general.

[B2] World Health Organization (2002). Prevention and promotion in mental health Geneva.

[B3] Health Canada (2001). A proactive approach to good health.

[B4] Sartorius N, Ustun TB, Lecrubier Y, Wittchen HU (1996). Depression comorbid with anxiety: Results from the WHO study on Psychological Disorders in Primary Health Care. British Journal of Psychiatry.

[B5] Nuyen J, Volkers AC, Verhaak PF, Schellevis FG, Groenewegen PP, Van den Bos GA (2005). Accuracy of diagnosing depression in primary care: the impact of chronic somatic and psychiatric co-morbidity. Psychol Med.

[B6] Ramasubbu R, Patten SB (2003). Effect of depression on stroke morbidity and mortality. Can J Psychiatry.

[B7] D'Alisa S, Miscio G, Baudo S, Simone A, Tesio L, Mauro A (2006). Depression is the main determinant of quality of life in multiple sclerosis: a classification-regression (CART) study. Disabil Rehabil.

[B8] Fruhwald S, Loffler H, Eher R, Saletu B, Baumhackl U (2001). Relationship between depression, anxiety and quality of life: a study of stroke patients compared to chronic low back pain and myocardial ischemia patients. Psychopathology.

[B9] Patten SB, Wang JL, Williams JV, Currie S, Beck CA, Maxwell CJ, El Guebaly N (2006). Descriptive epidemiology of major depression in Canada. Can J Psychiatry.

[B10] Offord DR, Boyle MH, Campbell D, Goering P, Lin E, Wong M, Racine YA (1996). One-year prevalence of psychiatric disorder in Ontarians 15 to 64 years of age. Canadian Journal of Psychiatry.

[B11] Patten SB (2002). Progress against major depression in Canada. Canadian Journal of Psychiatry.

[B12] Olfson M, Marcus SC, Druss B, Elinson L, Tanielian T, Pincus HA (2002). National trends in the outpatient treatment of depression. Journal of the American Medical Association.

[B13] Robins LN, Regier DA (1991). Psychiatric disorders in America: the Epidemiologic Catchment Area Study.

[B14] Ontario Ministry of Finance (2006). Ontario Demographic Quarterly.

[B15] Kessler RC, Mcgonagle KA, Zhao SY, Nelson CB, Hughes M, Eshleman S, Wittchen HU, Kendler KS (1994). Lifetime and 12-Month Prevalence of DSM-III-R psychiatric-disorders in the United-States – Results from the National-Comorbidity-Survey. Archives of General Psychiatry.

[B16] Patten SB (2000). Incidence of major depression in Canada. Canadian Medical Association Journal.

[B17] (2006). Australian Bureau of Statistics. http://www.abs.gov.au/Ausstats/abs@.nsf/Lookup/670D4F8706B05404CA2568A900136280.

[B18] Wade TJ, Cairney J, Pevalin DJ (2002). Emergence of gender differences in depression during adolescence: national panel results from three countries. J Am Acad Child Adolesc Psychiatry.

[B19] Kornstein SG, Schatzberg AF, Thase ME, Yonkers KA, McCullough JP, Keitner GI, Gelenberg AJ, Ryan CE, Hess AL, Harrison W (2000). Gender differences in chronic major and double depression. Journal of Affective Disorders.

[B20] Kornstein SG, Schatzberg AF, Thase ME, Yonkers KA, McCullough JP, Keitner GI, Gelenberg AJ, Davis SM, Harrison WM, Keller MB (2000). Gender differences in treatment response to sertraline versus imipramine in chronic depression. American Journal of Psychiatry.

[B21] Cairney J, Thorpe C, Rietschlin J, Avison WR (1999). 12-month prevalence of depression among single and married mothers in the 1994 National Population Health Survey. Canadian Journal of Public Health-Revue Canadienne de Sante Publique.

[B22] Eaton WW, Muntaner C, Bovasso G, Smith C (2001). Socioeconomic status and depressive syndrome: the role of inter- and intra-generational mobility, government assistance, and work environment. J Health Soc Behav.

[B23] Lennon MC, Blome J, English K (2002). Depression among women on welfare: A review of the literature. Depression among women on welfare: A review of the literature.

[B24] Lorant V, Deliege D, Eaton W, Robert A, Philippot P, Ansseau M (2003). Socioeconomic inequalities in depression: A meta-analysis. American Journal of Epidemiology.

[B25] Fortenberry JD (2003). Socioeconomic status, schools, and adolescent depression: Progress in the social epidemiology of adolescent health. Journal of Pediatrics.

[B26] Goode E (1997). Deviant Behavior.

[B27] Murphy JM, Olivier DC, Monson RR, Sobol AM, Federman EB, Leighton AH (1991). Depression and anxiety in relation to social status. A prospective epidemiologic study. Arch Gen Psychiatry.

[B28] Kohn R, Dohrenwend B, Mirotznik H, Dohrenwend B (1998). Edidemilogical findings on selected psychaitric disordrs in the general population. Adversity, stress and psychopathology.

[B29] Halter MJ (2004). The stigma of seeking care and depression. Archives of Psychiatric Nursing.

[B30] World Health Organization (2001). The World health report: Mental health: new understanding, new hope Geneva.

[B31] Karasz A (2005). Cultural differences in conceptual models of depression. Soc Sci Med.

[B32] Statistics Canada (2006). The Daily: Canada's population as of July 1, 2006 Ottawa.

[B33] Kessler RC, Ustun TB (2004). The World Mental Health (WMH) Survey Initiative Version of the World Health Organization (WHO) Composite International Diagnostic Interview (CIDI). International Journal of Methods in Psychiatric Research.

[B34] American Psychiatric Association (2000). Diagnostic and statistical manual of mental disorders.

[B35] Kessler RC, Abelson J, Demler O, Escobar JI, Gibbon M, Guyer ME, Howes MJ, Jin R, Vega WA, Walters EE (2004). Clinical calibration of DSM-IV diagnoses in the World Mental Health (WMH) version of the World Health Organization (WHO) composite international diagnostic interview (WMH-CIDI). International Journal of Methods in Psychiatric Research.

[B36] Statistics Canada (2006). Canada's population.

[B37] Gutierrez-Lobos K, Scherer M, Anderer P, Katschnig H (2002). The influence of age on the female/male ratio of treated incidence rates in depression. BMC Psychiatry.

[B38] Streiner DL, Cairney J, Veldhuizen S (2006). The epidemiology of psychological problems in the elderly. Can J Psychiatry.

[B39] Wade TJ, Cairney J (1997). Age and depression in a nationally representative sample of Canadians: a preliminary look at the National Population Health Survey. Can J PublicHealth.

[B40] Wade TJ, Cairney J (2000). The effect of sociodemographics, social stressors, health status and psychosocial resources on the age-depression relationship. Can J Public Health.

[B41] Kessler RC, Foster C, Webster PS, House JS (1992). The relationship between age and depressive symptoms in two national surveys. Psychol Aging.

[B42] Newmann JP (1989). Aging and depression. Psychol and Aging.

[B43] Scarinci IC, Beech BM, Naumann W, Kovach KW, Pugh L, Fapohunda B (2002). Depression, socioeconomic status, age, and marital status in black women: a national study. Ethn Dis.

[B44] Karasz A (2005). Cultural differences in conceptual models of depression. Soc Sci Med.

